# Phenolic plant extracts are additive in their effects against *in vitro* ruminal methane and ammonia formation

**DOI:** 10.5713/ajas.18.0665

**Published:** 2019-01-02

**Authors:** Susanne Sinz, Svenja Marquardt, Carla R. Soliva, Ueli Braun, Annette Liesegang, Michael Kreuzer

**Affiliations:** 1 Institute of Agricultural Sciences, ETH Zurich, Zurich 8092, Switzerland; 2 Department of Farm Animals, University of Zurich, Zurich 8057, Switzerland; 3 Institute of Animal Nutrition, Vetsuisse Faculty, University of Zürich, Zürich 8057, Switzerland

**Keywords:** Methane, Plant Extract, *Acacia mearnsii*, Grape Seed, Green Tea, *Uncaria gambir*

## Abstract

**Objective:**

The methane mitigating potential of various plant-based polyphenol sources is known, but effects of combinations have rarely been tested. The aim of the present study was to determine whether binary and 3-way combinations of such phenol sources affect ruminal fermentation less, similar or more intensively than separate applications.

**Methods:**

The extracts used were from *Acacia mearnsii bark* (acacia), *Vitis vinifera* (grape) seed, *Camellia sinensis* leaves (green tea), *Uncaria gambir* leaves (gambier), *Vaccinium macrocarpon* berries (cranberry), *Fagopyrum esculentum* seed (buckwheat), and *Ginkgo biloba* leaves (ginkgo). All extracts were tested using the Hohenheim gas test. This was done alone at 5% of dry matter (DM). Acacia was also combined with all other single extracts at 5% of DM each, and with two other phenol sources (all possible combinations) at 2.5%+2.5% of DM.

**Results:**

Methane formation was reduced by 7% to 9% by acacia, grape seed and green tea and, in addition, by most extract combinations with acacia. Grape seed and green tea alone and in combination with acacia also reduced methane proportion of total gas to the same degree. The extracts of buckwheat and gingko were poor in phenols and promoted ruminal fermentation. All treatments except green tea alone lowered ammonia concentration by up to 23%, and the binary combinations were more effective as acacia alone. With three extracts, linear effects were found with total gas and methane formation, while with ammonia and other traits linear effects were rare.

**Conclusion:**

The study identified methane and ammonia mitigating potential of various phenolic plant extracts and showed a number of additive and some non-linear effects of combinations of extracts. Further studies, especially in live animals, should concentrate on combinations of extracts from grape seed, green tea leaves Land acacia bark and determine the ideal dosages of such combinations for the purpose of methane mitigation.

## INTRODUCTION

Combating climate change requires suitable measures for mitigating greenhouse gas emissions like that of methane (CH_4_), a large share of which is contributed by agriculture. Dietary measures are important means for reducing CH_4_ emission from ruminants. A number of plant extracts rich in polyphenols and individual phenols have shown activity against CH_4_ formation [1]. Among the polyphenols, especially the tannins may protect dietary protein from ruminal degradation to ammonia [2] and thus help concomitantly reducing urinary N formation and, by this way, noxious N emissions like nitrous oxide and ammonia from the excreta [3]. Their natural origin makes phenols better accepted as feed additives by consumers than synthetic compounds. However, the balance between minimally effective and potentially anti-nutritional dosages is delicate. It may, therefore, be helpful to use more than one phenol source and thus individual sources at a lower dosage. Nevertheless, the efficiency of combinations of polyphenols, in comparison to their use as single additives, for reducing CH_4_ and ammonia emissions has rarely been investigated [4].

For this reason, the hypothesis that combinations of extracts rich in phenols will reduce ruminal CH_4_ and ammonia formation at least as efficient as the extracts alone, but this with less adverse side-effects on other ruminal fermentation parameters was tested *in vitro*. For that purpose, seven different phenolic extracts were tested alone or in combination with other extracts. Additivity was either tested by comparing the level of effect of binary combinations of extracts to the effect of acacia extract alone (with each single extract having the same dosage) or by comparing binary treatments with combinations with a third extract at the same total extract dosage.

## MATERIALS AND METHODS

### Animal care

Housing of, and rumen fluid collection from, the rumen cannulated cow was approved by the Zurich Cantonal Veterinary Office (licence no ZH 38/14).

### Test extracts

From the seven plant extracts investigated, three have been already investigated individually *in vivo* and *in vitro* for their CH_4_ mitigation properties. These were the extracts from *Acacia mearnsii* bark (acacia; e.g., AM [5,6]), *Camellia sinensis* leaves (green tea; GT) [7]) and *Fagopyrum esculentum* seeds (buckwheat; BW). For the latter plant, only effects of the whole grain or the whole plant were reported in the literature [8]. Furthermore, two extracts whose CH_4_ reducing potential has been tested *in vitro*, *Vitis vinifera* seeds (grape; GS [9]) and *Ginkgo biloba* leaves (ginkgo; GK [10]) were tested. Finally, two extracts were included that have not been investigated before for their CH_4_ mitigation potential, *Uncaria gambir* leaves (gambier; GB) and *Vaccinium macrocarpon* berries (cranberry; CB). Table 1 lists further details on the extracts used.

### Experimental treatments

Each extract was tested alone at a dosage of 5% in dry matter (DM) of a basal diet. Acacia (at 5%) was also tested in combination with each of the six other extracts (at 5%; total extract at 10% of DM). Finally, 5% of acacia was tested together with all possible combinations of two of the other six extracts, each provided at 2.5% in DM. This also added up to a total of 10% extract in DM like the binary combinations. The dosages were selected based on the following considerations: i) When testing the extracts alone the critical threshold of 5% of dietary phenols, especially tannins, should not be surpassed; going beyond may result more often in unfavorable side-effects like impairments of feed intake and rumen fermentation whereas for lower dosages often positive effects are reported. ii) As it was assumed that detrimental effects could be less pronounced when combining differently composed phenolic extracts, the extract dosage was doubled in these cases. The basal diet consisted of pure ryegrass (*Lolium multiflorum*) hay prepared from the first cut obtained from a local plant breeder. The ryegrass hay used in the experiment contained in DM: organic matter (OM), 93.9%; crude protein (CP), 5.1%; ether extract (EE), 1.5%; neutral detergent fiber (NDF), 61.8%; non-fiber carbohydrates (NFC), 36.7%; total extractable phenols (TEP), 0.15%. In the experiment, six different runs were carried out where each of the single test extract and each combination of test extracts were included (n = 6). In addition, a negative control (basal diet alone) was included in two replicates per run, with the values averaged per run for statistical analysis. This protocol resulted in a total of 180 incubations (30 per run).

### *In vitro* incubations

The batch culture Hohenheim gas test [16] was applied for the *in vitro* incubations. To allow for direct collection of fermentation gas samples, pistons modified with a second outlet were employed [16]. Rumen fluid was collected before morning feeding separately for each run during a period of 12 weeks from a rumen-cannulated lactating Brown Swiss cow. The rumen fluid had on average a pH of 7.1±0.3 and an ammonia concentration of 7.2±2.7 μmol/mL. The cow had *ad libitum* access to water. The diet consisted of ryegrass hay (the same hay was used as basal diet in the incubations) and grass silage (1:1). Additionally, the cow received 4 kg/d of a dairy concentrate (UFA Prima F 142, UFA AG, Sursee, Switzerland). After collection, the rumen fluid was strained through four layers of gauze. Menke buffer [16] was added in a ratio of 1:3 parts of rumen fluid adding up to 30 mL of incubation fluid per syringe. An amount of 200 mg DM of the basal diet, plus either 10 mg (one extract) or 20 mg (mixture of two or three extracts) of the respective test extracts were put together with the incubation fluid into the incubation syringes. Afterwards the syringes were incubated for 24 h in a rotor installed in a drying cabinet at 39°C.

After 24 h, the fermentation gas volume was read from the calibrated scale printed onto the syringe. Gas samples were taken using a sampling injector syringe through an airtight septum covering the second outlet. The CH_4_ concentrations were analyzed on a gas chromatograph (6890N, Agilent Technologies, Wilmington, DE, USA). Ammonia concentrations and pH (for control of sufficient buffering) of the incubation fluid were determined with a potentiometer (Model 632 and Model 713, Metrohm, Herisau, Switzerland) fitted with the respective electrodes. Short-chain fatty acids (SCFA) in incubation fluid were analyzed by high performance liquid chromatography (La Chrom, L-7000 series, Hitachi Ltd, Tokyo, Japan). For counting of total bacteria as well as total, holotrich, and entodiniomorph protozoa, Bürker counting chambers (Blau Brand, Wertheim, Germany) with a depth of either 0.02 mm (bacteria) or 0.1 mm depth (protozoa) were used. Diluted formaldehyde (0.04/L w/v in water) was used for fixing protozoa, while bacteria were fixed with Hayem solution (mmol/L: HgCl_2_, 9; Na_2_SO_4_, 176; NaCl, 86).

### Compositional analysis

Extracts and ryegrass hay were analyzed following standard protocols [17]. For DM and total ash analysis, a thermogravimetric device (model TGA 701, Leco Corporation, St Joseph, MI, USA; AOAC index no. 942.05) was used. The difference of DM and total ash is OM. A C/N-analyzer (TruMac CN, Leco Corporation, USA; AOAC index no. 968.06) was applied to determine N and CP was calculated as 6.25×N. Ether extract was analyzed with a Soxhlet extractor (extraction System B-811, Büchi, Flawil, Switzerland; AOAC index no. 963.15). The NDF content was determined with a Fibertec System M (Tecator, Höganäs, Sweden) using heat stable α-amylase but no sodium sulfite. Values were corrected by for ash content. The NFC were calculated as OM–CP–EE–NDF–TEP. For TEP and non-tannin phenol (NTP) analysis, the method applied by Jayanegara et al [18] was used which is based on a modified Folin-Ciocalteu method and gallic acid as a standard (Sigma, St. Louis, MO, USA). All values were expressed as gallic acid equivalents (Sigma, USA). Total tannins (TT) were calculated as the difference of TEP and NTP. The blue-dye bound bovine serum albumin (BSA) method was applied following a modified procedure of Asquith and Butler [19] to determine protein precipitation capacity. Briefly, 2 g BSA was mixed with 150 mg remazol brilliant blue R in 40 mL NaHCO_3_ solution (1%) and stirred for 30 min at room temperature. The pH was set to 4.8 using acetic acid. The solution was diluted to 250 mL using acetate buffer (pH 4.8). The single extracts, dissolved in methanol, were each mixed intensively with the blue dyed BSA solution (containing 4 mg blue BSA/mL) and centrifuged 10 min at 8,000×*g*. The supernatant was removed and 1 mL of isopropanol/sodium dodecyl sulfate/triethanolamine (200 mL/10 g/50 mL in 1 L of distilled water) was added to the precipitate and vortexed until complete dissolution. Photometric measurement was made at an absorbance of 590 nm using a UV-vis spectrophotometer (VWR UV-6300, VWR International, Radnor, PA, USA). The amount of protein precipitation was calculated from the calibration curve using different dissolutions of the blue dyed BSA.

### Statistical analysis

Data analysis was performed with the software R (R Development Core Team, Vienna, Austria). The lmer function was used to perform a mixed model with dietary treatment as fixed effect and run as random effect. Least square means of the extract treatments were statistically compared with the control treatment with Dunnett’s test. This model was used for analyzing all extract supplementations (single as well as combinations) in comparison to the control and for comparing the binary combinations with the acacia only treatment. The latter comparison tested whether the levels found with additional 5% of DM of another extract were different from those measured with acacia alone. Orthogonal polynomial contrast analysis was done to compare the 3-extract combinations (acacia plus two of the other extracts) with their two corresponding binary combinations. The aim of the latter analysis was to identify linear effects (i.e., the value of the 3-extract combination is similar to the mean value of the corresponding binary combinations) and non-linear effects (here: quadratic, i.e., the value of the 3-extract combination differs from the mean value of the corresponding binary combinations). The boxplots displayed in the figures were created with the R software (R Development Core Team, Austria).

## RESULTS

### Composition of the experimental extracts

The extracts prepared from acacia bark, grape seed, green tea leaves, gambier leaves, and cranberry berries had TEP concentrations of ≥40% in DM, whereas the extracts from buckwheat seed and ginkgo leaves were rather low in phenols (Table 2). The TT made up the majority (88% to 96%) of the TEP in the extracts rich in phenols. The protein precipitation capacity of the extracts closely corresponded to the concentrations of TEP and TT, except for cranberry where this capacity was proportionately higher than expected. The extracts low in TEP content had a high NFC content. Concerning CP, the extracts of green tea (8% DM) and gambier (18% DM) were higher than the other extracts (≤3% DM). The concentrations of TEP with the phenol-rich extracts ranged from 2.0 (cranberry) to 4.0% (grape seed) of dietary DM and those of TT from 1.7% to 3.7% of DM, respectively, when 5% single extracts were added. In case of the 10% dosages, the corresponding ranges (in % of dietary DM) were from 4.6 (acacia+cranberry) to 6.6 (acacia+grape seed) and from 4.0 to 6.0, respectively. The extracts from buckwheat and ginkgo only provided minimal levels.

### Effects of the extracts alone and in combination on total fermentation gas and methane

**Figure 1:**
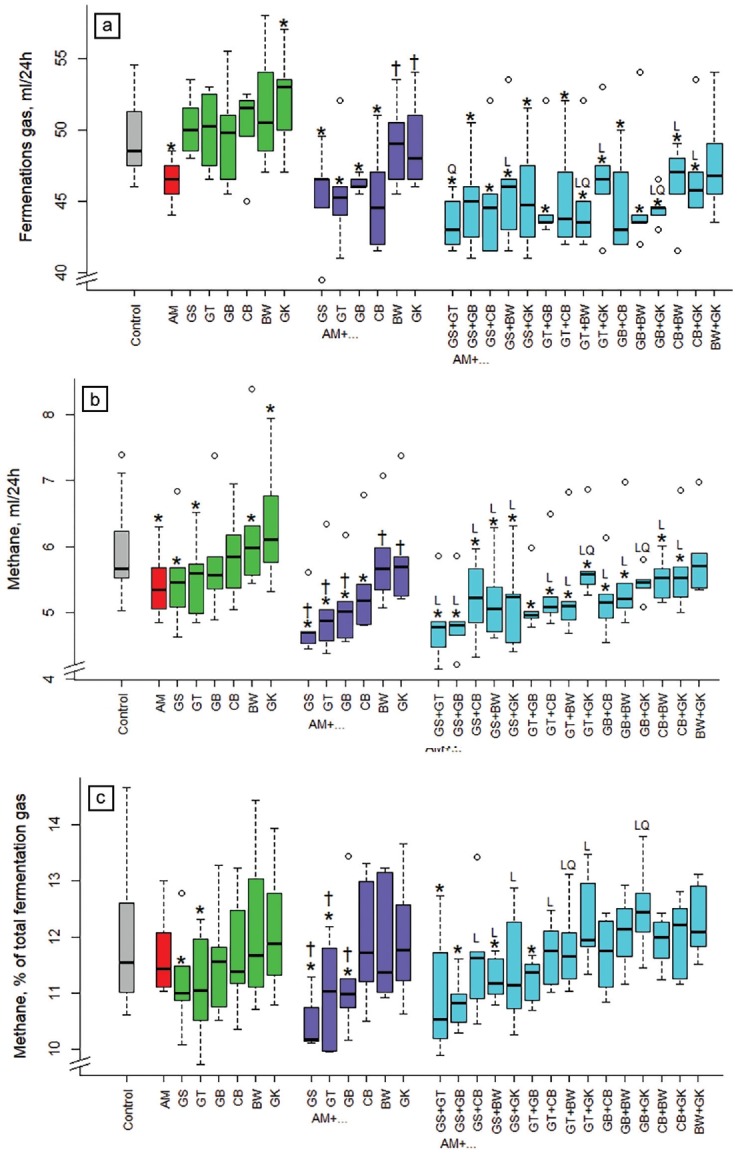
Influence of different plant extracts (AM, *Acacia mearnsii* bark; GS, grape seed; GT, green tea leaves; BW, buckwheat seed; GK, gingko leaves; GB, gambier leaves; CB, cranberry berries) alone or in combination with AM on *in vitro* production of (a) fermentation gas and (b) of methane during 24 h, as well as (c) methane in % of total gas. Dosages: alone: 5% extract per DM; binary combinations: 5%+5% extract per DM; combinations of three extracts: 5% AM+2.5%+2.5% per DM. DM, dry matter. * Means of boxplots differ (p<0.05) from the control without extract supplementation. ^†^ Means of boxplots differ (p<0.05) from AM only treatment. L, linear contrast; Q, quadratic contrast; significant at p<0.05 when relating results of the 3-way combinations to the corresponding binary combinations.

Compared to the non-supplemented control diet, total fermentation gas production was reduced (p<0.05) by 6% with acacia alone and by 8% on average in all combinations with acacia, except those with buckwheat and gingko alone or in combination (BW+GK) (Figure 1a). When comparing the combination of three extracts (acacia plus two other extracts) with their corresponding binary combinations, the reduction in total fermentation gas production was linear (p<0.05) with GS+BW, GT+GK, CB+BW, CB+GK, quadratic (p<0.05) with GS+GT, and both linear and quadratic (p<0.05) with GT+BW and GB+GK. Total gas production with ginkgo and buckwheat alone was higher (p<0.05 and p<0.01) when compared to the control. Binary combinations of gingko and buckwheat with acacia increased (p<0.05) fermentation gas production compared to acacia alone.

Methane production was reduced (p <0.05) compared to the control by acacia alone and all combinations with acacia, except for the binary and 3-way combinations with buckwheat and ginkgo as well as the 3-way combination with gambier and ginkgo (Figure 1b). Binary combinations of grape seed, green tea, and gambier with acacia were more effective (p<0.05) than acacia alone. The reduction of CH_4_ formation was mostly linear (p<0.05) with the 3-way combinations to the corresponding binary combinations, except with GT+GK and GB+GK where the relationship was both linear and quadratic (p<0.05), and with GT+GB and BW+GK were no linear or quadratic relationships were found. Compared to the control, the CH_4_ formation increased (p<0.05) when supplementing ginkgo and buckwheat alone and when comparing acacia alone with its binary combinations with buckwheat and ginkgo.

Compared to the control, the CH _4_ proportion of total fermentation gas was reduced (p<0.05) by −7.1% and −7.2% with the extracts from grape seed and green tea, respectively (Figure 1c). This ratio was also reduced (p<0.05) with binary combinations of acacia and GS or GT (and AM+GB) and with several of its 3-way combinations (AM+GS+GT, AM+GS+GB, AM+ GS+BW, AM+GT+GB). Binary combinations of grape seed, green tea, and gambier with acacia were more effective (p<0.05) than acacia alone. The reduction of the CH_4_ proportion of total fermentation gas was linear (p<0.05) when combining GS+CB, GS+BW, GS+GK, GT+CB, GT+GK, with acacia relative to the respective binary combinations, and was both linear and quadratic (p<0.05) with GT+BW and GB+GK.

### Effects of the extracts alone and in combination on ammonia and microbial counts

**Figure 2:**
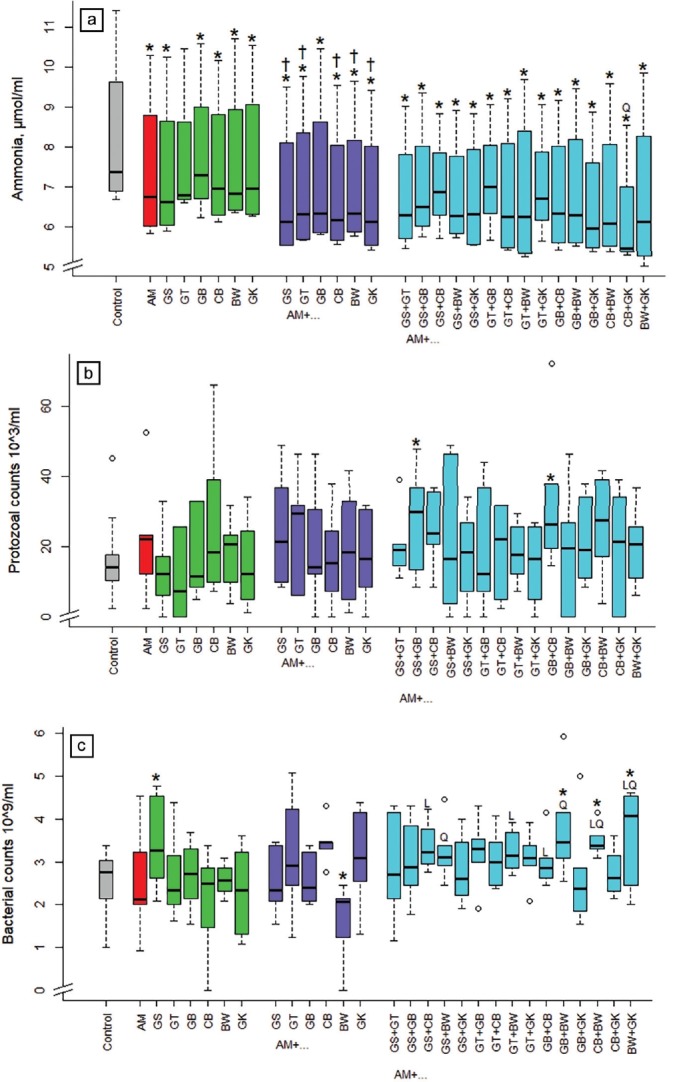
Influence of different plant extracts (AM, *Acacia mearnsii* bark; GS, grape seed; GT, green tea leaves; BW, buckwheat seed; GK, gingko leaves; GB, gambier leaves; CB, cranberry berries) alone or in combination with AM on ammonia concentration (a), protozoal (b), and bacterial counts (c) in incubation fluid, measured after 24 h of fermentation *in vitro*. Dosages: alone: 5% extract per DM; binary combinations: 5%+5% extract per DM; combinations of three extracts: 5% AM+2.5%+2.5% per DM. DM, dry matter. * Means of boxplots differ (p<0.05) from the control without extract supplementation. ^†^ Means of boxplots differ (p<0.05) from AM only treatment. L, linear contrast; Q, quadratic contrast significant at p<0.05 when relating results of the 3-way combinations to the corresponding binary combinations.

All single extract treatments, except green tea when provided alone, lowered ammonia concentration in the incubation fluid in comparison with the control (Figure 2a) by on average 15%. Combinations with grape seed, green tea, cranberry, buckwheat, and ginkgo were more effective (p<0.05) than acacia alone. The combination of cranberry and ginkgo with acacia was in between the binary combinations in a quadratic (p<0.05) manner. The protozoal counts were increased (p<0.05) when adding the 3-way combinations of GS+GB and GB+CB with acacia by 72% and 104%, respectively (Figure 2b). Bacterial counts were increased (p<0.05) when incubating grape seed alone and in some 3-way combinations including buckwheat (AM+GB+BW, AM+CB+BW, AM+BW+GK; Figure 2c). The binary combination of acacia and buckwheat lowered (p<0.05) bacterial counts by 36%. The 3-way-combination was linear (p<0.05) in between the respective binary combinations with GS+CB, GT+BW, and GB+CB, quadratic (p<0.05) with GS+ BW and GB+BW, and linear and quadratic (p<0.05) with CB+ BW, BW+GK.

### Effects of the extracts alone and in combination on short-chain fatty acids

**Figure 3:**
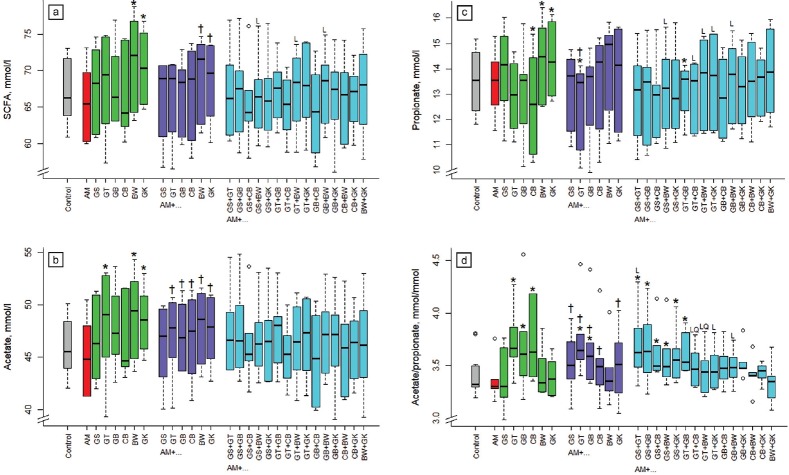
Influence of different plant extracts (AM, *Acacia mearnsii* bark; GS, grape seed; GT, green tea leaves; BW, buckwheat seed; GK, gingko leaves; GB, gambier leaves; CB, cranberry berries) alone or in combination with AM on total short-chain fatty acid (SCFA) concentration (a), acetate concentration (b), propionate concentration (c) and acetate/propionate ratio (d) in incubation fluid, measured after 24 h of fermentation *in vitro*. Dosages: alone: 5% extract per DM; binary combinations: 5%+5% extract per DM; combinations of three extracts: 5% AM+2.5%+2.5% per DM. DM, dry matter. * Means of boxplots differ (p<0.05) from the control without extract supplementation. ^†^ Means of boxplots differ (p<0.05) from AM only treatment. L, linear contrast; Q, quadratic contrast significant at p<0.05 when relating results of the 3-way combinations to the corresponding binary combinations.

Supplementing buckwheat and ginkgo alone increased (p<0.05) SCFA concentration in incubation fluid compared to the control (Figure 3a) by 5% to 6%. Binary combinations of buckwheat and ginkgo with acacia increased SCFA concentration more (p<0.05) than acacia alone. The increase found with the 3-way-combination was linear (p<0.05) with AM+GS+BW, AM+GT+ BW, and AM+ GB+ BW.

Relative to the control, acetate concentration in incubation fluid increased (p<0.05) when supplementing green tea, buckwheat, and ginkgo alone (Figure 3b) by 4% to 6%. Compared to acacia alone, acetate concentration increased in the binary combinations with green tea, gambier, cranberry, buckwheat, and ginkgo. Propionate concentration in incubation fluid was increased (p<0.05) by 6% with the supplementation of buckwheat and ginkgo alone (Figure 3c). Supplementing cranberry alone and acacia with green tea and with green tea and gambier reduced (p<0.05) propionate concentration compared to the control, by 6% to 7%. The relationship for propionate concentration compared to the binary combination was linear (p<0.05) when combining acacia with GS+BW, GT+CB, GT+ BW, GT+GK, and GB+BW.

The ratio of acetate to propionate in incubation fluid was increased (p<0.05) by green tea, gambier, and cranberry alone and by combinations of acacia with GT, GB, GS+GT, GS+GB, GS+CB, GS+BW, GS+GK, or GT+GB (Figure 3d). The relationship compared to the binary combinations was linear (p< 0.05) with acacia and GS+GT, GT+GK, GB+BW, and linear and quadratic (p<0.05) with GT+CB and GT+BW.

**Figure 4:**
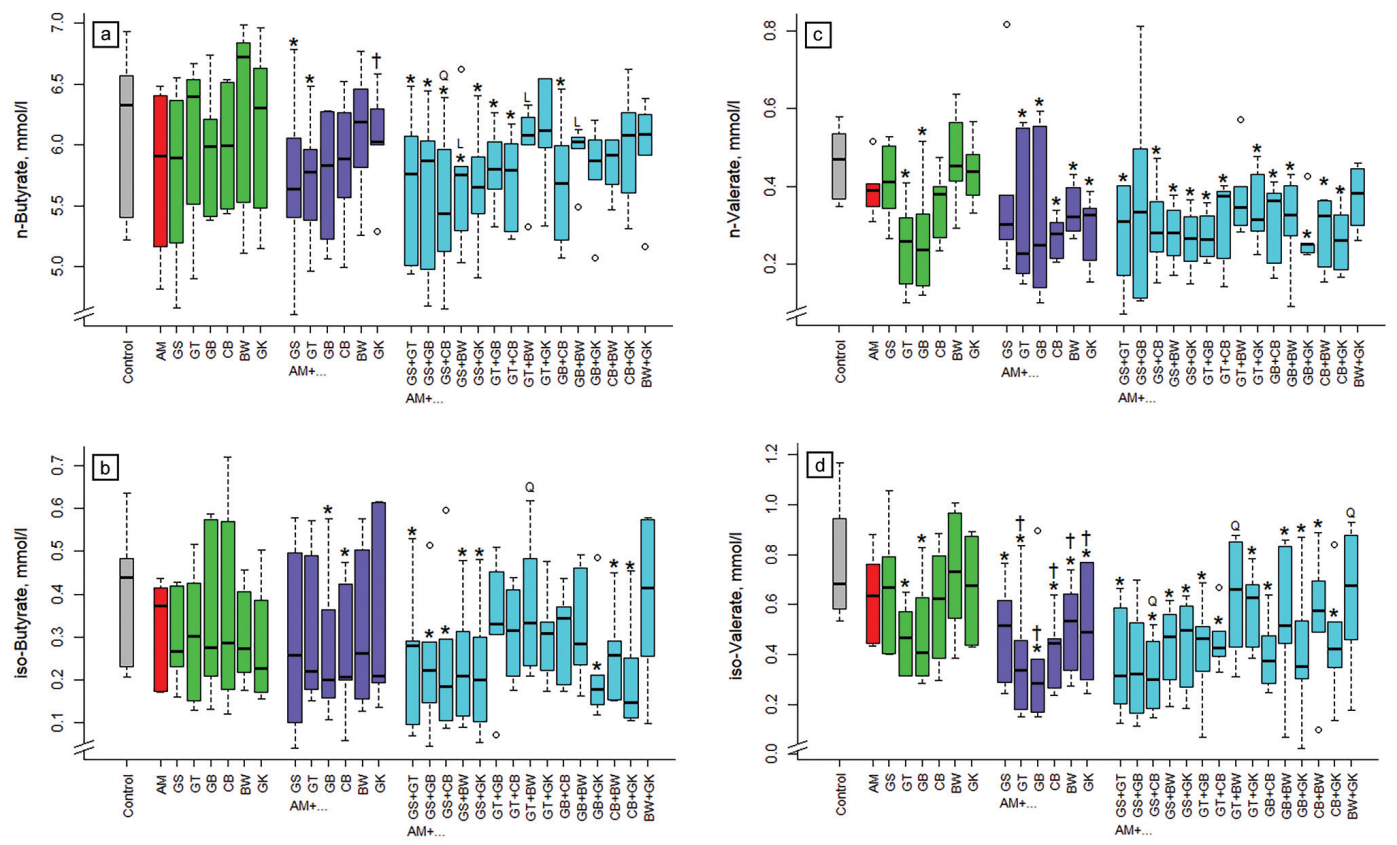
Influence of different plant extracts (AM, *Acacia mearnsii* bark; GS, grape seed; GT, green tea leaves; BW, buckwheat seed; GK, gingko leaves; GB, gambier leaves; CB, cranberry berries) alone or in combination with AM on concentrations of *n*-butyrate (a), *iso*-butyrate (b), *n*-valerate (c), and *iso*-valerate (d) in incubation fluid, measured after 24 h of fermentation *in vitro*. Dosages: alone: 5% extract per DM; binary combinations: 5%+5% extract per DM; combinations of three extracts: 5% AM+2.5%+2.5% per DM. * Means of boxplots differ (p<0.05) from the control without extract supplementation. ^†^ Means of boxplots differ (p<0.05) from AM only treatment. L, linear contrast; Q, quadratic contrast significant at p<0.05 when relating results of the 3-way combinations to the corresponding binary combinations.

None of the single extracts affected *n*-butyrate concentration in incubation fluid (Figure 4a). Some of the acacia combinations reduced (p<0.05) *n*-butyrate concentration, namely those with GS, GT, and with GS+GT, GS+GB, GS+CB, GS+BW, GS+GK, GT+GB, GT+CB, GB+CB. The binary combination of ginkgo with acacia increased *n*-butyrate concentration (p< 0.05) compared to acacia alone. The relationship of the 3-way combinations compared to the binary combinations was linear (p<0.05) with GS+BW, GT+BW, and GB+ BW, and quadratic (p<0.05) with GS+CB. The concentration of *iso*-butyrate was reduced (p<0.05) by combinations of acacia with gambier, cranberry, GS+GT, GS+GB, GS+CB, GS+BW, GS+GK, GB+GK, CB+BW, or CB+GK (Figure 4b). The relationship was quadratic (p<0.05) for AM+GT+BW.

The concentration of *n*-valerate in incubation fluid was reduced (p<0.05) by green tea and gambier alone and most of the binary and 3-way combinations (except: AM+GS, AM+ GS+GB, AM+GT+BW, AM+BW+GK; Figure 4c). The *iso*-valerate concentration was also reduced (p<0.05) by most of the binary and 3-way combinations (except: AM+GS+GB, AM+GT+BW, AM+BW+GK). The binary combinations of green tea, gambier, and cranberry with acacia decreased (p< 0.05) *n*-butyrate compared to acacia alone. Concerning *iso*-valerate, the relationship was quadratic (p<0.05) for AM+GS+ CB, AM+GT+BW, and AM+BW+GK.

## DISCUSSION

### Effects of the extracts from buckwheat seeds and ginkgo leaves

Among the large number of extracts available and expected to be effective against ruminal CH_4_ or ammonia formation, only some turn out to be effective. Others may not have these mitigating properties but may be found to possess a nutritional value. A similar phenomenon occurred in the present study with extracts from buckwheat seeds and ginkgo leaves. These two were found to have rather low concentrations of TEP and TT, a finding which deviated largely from the providers’ specifications and therefore largely differed from the other five extracts tested. This was the likely main reason for the contrasting effects of these two extracts vs the others. Concomitant with the low phenol concentration, these two extracts were rich in nutrients (NFC; i.e. either starch or sugar) and thus were able to promote the production of fermentation gas and SCFA production (estimated by the concentration after 24 h of fermentation) and thus the amount of fermentable OM when they were added on top of the basal diet. The high level of NFC (>88%) in these two extracts also promoted the formation of all major SCFA and did not change the acetate/propionate ratio suggesting a basically unchanged fermentation profile. On a first glance it seemed puzzling that both extracts were still able to significantly reduce ammonia formation even though their protein precipitation capacity was low. However, the extra fermentable OM may have promoted the incorporation of ammonia N into microbial N. Due to their low TEP content and the deviating influence, the results obtained with the buckwheat and ginkgo extracts in the present study are not further discussed in the following in comparison with the other extracts.

### Comparative effects of the five plant extracts rich in phenols

In the present study, three of the five extracts with a TEP content of ≥40% in DM, namely acacia, grape seed, and green tea, were found to reduce absolute CH_4_ emission. The CH_4_ mitigation property of *Acacia mearnsii* bark has already been reported from several experiments, including several *in vivo* studies [e.g., 5,20]. This had been the reason for using acacia as a positive control and as the extract being present in all binary and 3-way combinations in the present study. Along with mitigating absolute CH_4_ formation when provided alone and in combination with other extracts, acacia concomitantly reduced fermentation gas production but not SCFA production (estimated by the final SCFA concentration in the incubation fluid). Similarly, Junior et al [21] found no influence of 0.6% of the acacia extract on SCFA concentration but a reduction in CH_4_ production in an e*x situ* ruminal fermentation study. Hassanat and Benchaar [6] also found that 5% *Acacia mearnsii* extract simultaneously reduced CH_4_ and fermentation gas volume *in vitro* but, different from the present study, also SCFA production was reduced. The reduction in ruminal fermentation (total gas and, numerically, SCFA production) by acacia found in the present experiment could be due to a direct inhibition of the rumen microorganism or their enzyme activity or both [22], but also due to binding of the acacia phenols to dietary fiber or protein, making the latter two partially inaccessible for microbial degradation [23]. Fermentation gas and SCFA production are indicators of energy (net energy for lactation and metabolizable energy) content of the diet [16]. The ratio of CH_4_/total gas is therefore an indicator if there is indeed a mitigation in the CH_4_ yield, which could translate into lower emissions per unit meat or milk (emission intensity). With the acacia extract, there was obviously no such CH_4_ mitigation in the present experiment.

Different from acacia, the two other effective extracts, grape seed, and green tea, were able to mitigate also the CH_4_ proportion of total fermentation gas, as they did not concomitantly impair production of total fermentation gas and SCFA. Wischer et al [9] and Pellikaan et al [24] also found no influence on total fermentation gas and SCFA formation but a reduction of CH_4_ production *in vitro* when adding grape seed extract at dosages between 3% and 12% in DM. No influence of grape seed extract and green tea extract on either total fermentation gas and SCFA or CH_4_ formation were reported Pellikaan et al [24] at dosages similar or higher than those tested in the present study. Different from that, Aemiro et al [7] found a reduction of CH_4_ formation with 4% green tea extract but, concomitantly, also a reduction in SCFA concentration. Since the CH_4_ mitigating effect of grape seed and green tea was not accompanied by a lower acetate formation (indicated by acetate concentration after fermentation) and acetate/propionate ratio it can be ruled out that the tannins reduced fiber degradation and thus did not indirectly inhibit CH_4_ formation. As also protozoal count remained constant, the phenols in these two extracts seem to directly inhibit the ruminal methanogens [22].

The two further extracts rich in phenols, gambier, and cran berry, exhibited no CH_4_ mitigation potential in the present study. To the knowledge of the authors, these two plant extracts have not been investigated before in this respect. The differences in effect between extracts provided at similar TEP dosages can be related to chemical structure, such as monomeric compounds, and polymer size, which influences the CH_4_ mitigating properties for instance of tannins [25]. The plant parts where the extracts originated from greatly differed in their main phenolic compounds (Table 1). Some of these compounds (epicatechin and quercetin) were found to be able to mitigate CH_4_ formation *in vitro* when provided alone [1].

Apart from the CH _4_ mitigating properties, an important effect of polyphenols may consist in reducing the ruminal protein degradation to ammonia and thus enhancing duodenal flow of rumen-undegradable protein. The protein precipitation capacity, an indicator of the protein protection activity of phenols, was much higher in all extracts rich in TEP compared to those with low TEP content. Accordingly, the supplementation of extracts from acacia, grape seed, gambier, and cranberry significantly lowered ammonia concentration in incubation fluid. One exception was green tea. However, the decline with green tea in this variable was numerically as high as that of the other four extracts and a higher individual variation may have prevented reaching significance. The ammonia mitigation properties of the extracts from acacia, grape seed, and green tea have been tested in other *in vitro* studies. For instance, Hassanat and Benchaar [6] described a reduction of ammonia concentration by 47% *in vitro* with a dosage of 5% in DM of an acacia extract. Pellikaan et al [24] reported reductions in ammonia by 40% and 22% *in vitro*, respectively, with 10% of grape seed and green tea extracts, respectively. Aemiro et al [7] and Wischer et al [9] reported lower reductions (8% to 9%) in ammonia concentration *in vitro* by either 10% grape seed extract or 5% green tea extract. In contrast to this, Junior et al [21] found no influence on ammonia concentration when testing the acacia extract *in vitro*. Apart from the protein-binding activity, other properties of polyphenols, like an adverse influence on either proteolytic bacteria or protease activity [26] or both, may decrease ruminal protein degradation further.

### Effects of adding a second plant extract to the acacia bark extract

One aim of the present study was to test whether the potential for CH_4_ and ammonia mitigation was already fully exhibited when adding 5% of an effective extract (here: acacia) or if additional 5% of another extract may enhance the effects. Concerning CH_4_ production, additional effects were found for grape seed, green tea, and gambier, but not for cranberry. Indeed, the addition of grape seed, green tea, and gambier to acacia reduced the CH_4_ proportion of total fermentation gas 5, 4, and 3 times more, respectively, than the level of reduction found when supplementing only acacia. This indicates that the effects of the binary combinations may be synergistic. Also, ammonia concentration was further reduced when combining acacia with all extracts except green tea, but this reduction was on average only 1.5 times greater than that caused by acacia alone indicating that effects were not fully additive. One of the few other indications given for the additivity of effects of two different extracts can be derived from comparing several *in vitro* studies investigating either single extracts of quebracho (up to 50 g/kg DM [e.g., 27]) and chestnut (36 g/kg DM [28]) or a combination of these two extracts (18 g/kg DM [29]). The extracts given alone had no effect, whereas the combination decreased methanogenensis. Overall, this shows that combining two phenol rich extracts can enhance their CH_4_ mitigating effect.

### Linearity and non-linearity of the effects of combinations of three extracts

A second aim of the present study was to find out whether the same dosage of combinations of two extracts in addition to acacia act similarly (linear; equal to the average) or whether they have even stronger or weaker (quadratic) effects than when the extracts were combined individually with acacia. In the formation of fermentation gas and CH_4_, most of the three extract combinations that significantly differed from the control, had exhibited linear and, less frequently, a quadratic relationship to their corresponding two extract-combinations. For a number of other parameters (ammonia concentration, microbial counts, concentrations of total and individual SCFA), the relationship between the three extract-combinations with their corresponding 2-extract-combinations was mostly not linear. There are three *in vitro* studies regarding the influence of mixtures of forages rich in tannins on fermentation parameters, CH_4_ and ammonia formation. Seresinhe et al [30] tested *Terminalia catappa*, *Acacia auriculiformis*, *Calliandra calothyrsus*, *Symplocos splicata*, *Mangifera indica*, and S*yzygium caryophyllatum* and Hess et al [31] tested the tannin-rich legume *Calliandra calothyrsus* (from two different countries) and the low-tannin legume *Cratylia argentea* alone or in combination. Cieslak et al [4] evaluated *in vitro* the influence of 0.25% of tannins from *Quercus cortex* or *Vaccinium vitis idaea* alone or in combination. None of these researchers specifically tested for linearity or non-linearity of these effects. The CH_4_ formation data (mmol/d and mmol/OM degraded) described in Hess et al [31] indicate that the values found with the combination of the legumes were very close to the average of the single legume supplementation, i.e., linear. The same is true for the reduction in absolute CH_4_ formation found by Cieslak et al [4] when combining *Quercus cortex* and *Vaccinium vitis idaea*. However, concerning CH_4_ in relation to total fermentation gas the deviation from the average was about 13% indicating that the supplements were not fully additive in that study. In the present study, it seems that, at a dosage of 10% in DM, adding a third extract counteracted the effects of the other extracts rather than promoting them. This points towards an interaction of the different phenols in the different extracts or with other dietary constituents or both. Cieslak et al [4] also suggested the presence of an interaction when comparing the effect of incubating the roots of *Saponaria officinalis* with the effect of an extract of the same root.

## IMPLICATIONS

Grape seed extract was superior to all others in reducing methane and ammonia emission *in vitro* without adversely influencing rumen fermentation. However, green tea extract was almost as effective, whereas the acacia extract concomitantly reduced ruminal nutrient fermentation. Additivity or partial additivity of effects was given for a number of variables and combinations of extracts but using more than two extracts seems not to be advantageous. Binary combinations to avoid using high dietary proportions of single extracts, thereby reducing the risk of palatability problems. Studies in live animals have to confirm the efficiency of the most promising binary combinations.

## Figures and Tables

**Table 1:** Description of the plant extracts used in the experiment

Plant species (trivial name)	Plant part	Type of extraction	Provider	Specification by provider	Main phenols	Reference
*Acacia mearnsii* (acacia; Weibull black)	Bark	W	CDM GmbH, Hamburg, Germany	72% tannins	Catechin, gallocatechin, robinetinidol fisetinidol	[11]
*Vitis vinifera* (grape; OmniVin20R)	Seed	W and E	Ajinomoto OmniChem Natural Specialities, Wetteren, Belgium	95% polyphenols	Gallic acid, catechin, epicatechin, epicatechin gallate	[12]
*Camellia sinensis* (green tea)	Leaf	W and E	Nanjing Zelang Medical Technology, Nanjing, China	95% polyphenols	Epicatechin, epicatechin-3-gallate, epigallocatechin, epigallocatechin-3-gallate	[13]
*Uncaria gambir* (gambier)	Leaf	W and E	Nanjing Zelang Medical Technology, Nanjing, China	50% catechin	Gambirin, catechin, procyanidin	[14]
*Vaccinium macrocarpon* (cranberry)	Berry	W and E	Changsha Zhonren Bio-technology, Changsha, China	50% OPC5	Kaempferol, quercetin and anthocyanin-type compounds	[15]
*Fagopyrum esculentum* (buckwheat)	Seed	W and E	Changsha Zhonren Bio-technology, Changsha, China	50% flavanoids	Contained only very low amounts of phenols (see Table 2)	
*Ginkgo biloba* (gingko)	Leaf	W and E	Nanjing Zelang Medical Technology, Nanjing, China	24% flavones	Contained only very low amounts of phenols (see Table 2)	

W, water; E, ethanol; OPC5, oligomere proanthocyanidine.

**Table 2:** Chemical composition (% of DM; DM, % of original matter) and protein precipitation capacity (mg BSA/mg sample) of the plant extracts used in the experiment

Extract type (trivial name)	DM	OM	CP	EE	NDF	NFC1)	TEP2)	TT2)	PPC
*Acacia mearnsii* bark (acacia)	91.3	95.5	3.03	0.17	0.85	40.1	51.3	46.5	8.53
*Vitis vinifera* seed (green tea)	92.8	98.3	0.43	0.04	0.55	16.6	80.7	74.3	7.38
*Camellia sinensis* leaf (grape seed)	95.8	99.9	7.89	0.06	0.34	13.9	77.7	74.3	7.90
*Uncaria gambir* leaf (gambier)	96.0	98.4	18.24	0.08	0.91	19.0	60.2	56.4	4.37
*Vaccinium macrocarpon* berry (cranberry)	94.5	98.9	1.88	0.11	0.81	56.4	39.7	35.0	6.63
*Fagopyrum esculentum* seed (buckwheat)	93.2	95.8	2.33	0.13	0.70	87.6	5.1	1.9	0.82
*Ginkgo biloba* leaf (ginkgo)	94.4	99.5	1.89	0.04	0.54	95.8	1.2	0.4	1.36

DM, dry matter; BSA, bovine serum albumin; OM, organic matter; CP, crude protein; EE, ether extract; NDF, neutral detergent fiber; NFC, non-fiber carbohydrates; TEP, total extractable phenols. TT, total tannins; PPC, protein precipitation capacity.

1) Calculated as NFC=OM–CP–EE–NDF–TEP.

2) Given as gallic acid equivalents.
